# CBPR Partnerships and Near-Roadway Pollution: A Promising Strategy to Influence the Translation of Research into Practice

**DOI:** 10.3390/environments7060044

**Published:** 2020-06-10

**Authors:** Linda S. Sprague Martinez, Wig Zamore, Alex Finley, Ellin Reisner, Lydia Lowe, Doug Brugge

**Affiliations:** 1 School of Social Work, Macro Department, Boston University, 264 Bay State Road, Boston, MA 02215, USA; 2 Somerville Transportation Equity Partnership, Somerville, MA 02145, USA; 3 Department of Public Health and Community Medicine, Tufts University, Medford, MA 02155, USA; 4 Chinatown Community Land Trust, Boston, MA 02111, USA; 5 Department of Public Health Sciences, University of Connecticut School of Medicine, Farmington, CT 06032, USA

**Keywords:** community-based participatory research, translation into policy and practice, traffic-related air pollution, transportation, transportation planning

## Abstract

Community-based participatory research (CBPR) aims to engage those traditionally left out of the research process. Partnering with community stakeholders to design, plan, implement and disseminate research can facilitate translation into practice. Using qualitative research methods, we set out to explore the policy and practice implications of a CBPR partnership focused on reducing exposure to near-roadway pollution. Key Informant interviews (*n* = 13) were conducted with individuals from various entities (municipal, state and private) for whom partners to the Community Assessment of Freeway Exposure and Health (CAFEH) provided technical assistance between 2013 and 2017. The findings indicate community research partnerships may have the power to inform local planning efforts. Developers and planners who the partnership consulted indicated a greater awareness of the implications of near-roadway exposure. They also described making changes in their practice based on study findings. The CAFEH partnership has demonstrated active attention to translating knowledge can influence local planning and practice, albeit with some challenges.

## 1. Introduction

Community-based participatory research partnerships (CBPR) aim to engage those traditionally left out of the research process and involve both researchers and community stakeholders working collaboratively [1–4]. Engaging community stakeholders in the research process, from planning and implementation to dissemination, is intended to facilitate translational research efforts by creating a common way of talking about and understanding research [5], deciphering the science so that it can be incorporated into practice and policy settings. We set out to explore how knowledge generated through CBPR partnerships may influence local planning, practice and policy.

A brief background on near-roadway pollution is provided, along with a description of our partnership. The methods are then outlined, followed by the findings and a discussion of the implications. This paper adds to the literature by exploring how research findings were used to inform local-level efforts related to near-roadway pollution. The findings indicate community research partnerships can bridge the academy and community divide, providing a pathway for knowledge translation. However, evidence of partnership contributions to actual policy change was not present.

### 1.1. Near Roadway Exposure and Health

There is convincing evidence that living within 100–200 m of major roadways is associated with adverse health, including respiratory, cardiovascular and neurological outcomes [6,7]. A variety of factors, including air pollutants and noise, are elevated next to roadways with high levels of traffic. While the relative effects of these factors are still being determined, there is evidence each may contribute to negative health outcomes [8]. As evidence that elevated air pollution from traffic as a risk factor has mounted, so too have attempts to reduce the risk of exposure.

**Figure 1.**
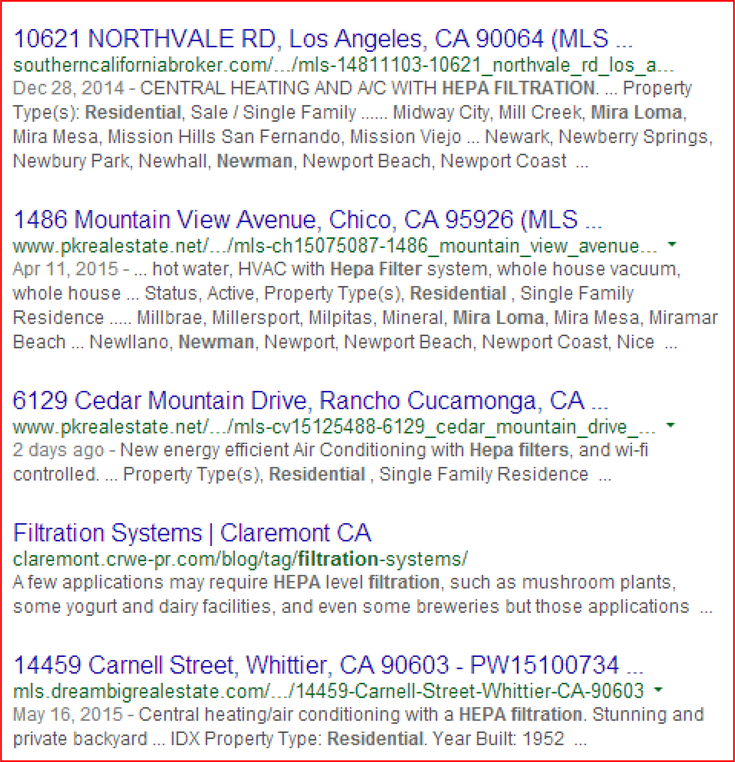
California Real estate advertisements highlighting HEPA filtration as an feature.

California is the only US state that has taken policy initiatives on these issues. For example, the state restricted building new schools within 500 feet of freeways [9], and the City of Los Angeles (L.A.) has recently required high-quality filtration in ventilation systems in residential buildings next to highways [10]. Meanwhile, at the individual level there has been increased interest in the purchase of stand-alone in-home air filters [11]. Real-estate advertisements, as demonstrated in Figure 1, indicate home brokers and developers in southern California see a market for enhanced filtration. Figure 1 is a series of 2014–2015 listings from the L.A. area. Each advertisement highlights in-home high-efficiency particulate air (HEPA) filtration as a feature.

On a broader scale, new energy codes require mechanical ventilation systems and therefore filtration, albeit not of high-grade filters. For example, the International Energy Conservation Code (IECC) 2015 and IECC 2018 require mandatory mechanical ventilation in order to assure fresh air supply in tighter buildings.

In the context of growing concerns about exposure to pollution for people who live, recreate and work near highways, and with the increasing number of vehicles that travel them daily in recent years, it is important to better understand how diffusion of knowledge of risk and possible responses occur. CBPR partnerships that join university researchers with local leaders from across multiple sectors of community life, including community-based organizations, government agencies and elected officials, may provide an effective vehicle for promoting sustainable change. Given successes on the west coast, we set out to explore the extent to which knowledge generated by our CBPR partnership in Massachusetts was being translated locally.

### 1.2. Community Assessment of Freeway Exposure and Health (CAFEH)

Community Assessment of Freeway Exposure and Health (CAFEH) is a CBPR partnership that was initiated by community leaders in the City of Somerville, Massachusetts. Somerville, which is adjacent to Boston, is the most densely populated city in New England, with over 80,000 residents living in just 4 square miles [12]. Over a decade ago, community activists in Somerville approached researchers with their concerns about near-roadway air pollution. Initially, it was proposed that university researchers provide technical consultation for a lawsuit the action group was proposing. What emerged, however, was a plan to design a collaborative research study to further examine the relationship between near-roadway pollution and health [13,14]. In 2008, the partnership received a CBPR grant from the National Institute of Environmental Health Sciences (NIEHS) to test associations between exposure to ultrafine particles (UFPs) near highways and cardiovascular disease risk in older adults in Somerville and Boston. The Chinatown neighborhood of Boston was included in the grant because of its proximity to the highway and because study investigators had strong relationships within the community. The Chinatown partners joined the study based on their concern for the health of community residents.

**Figure 2.**
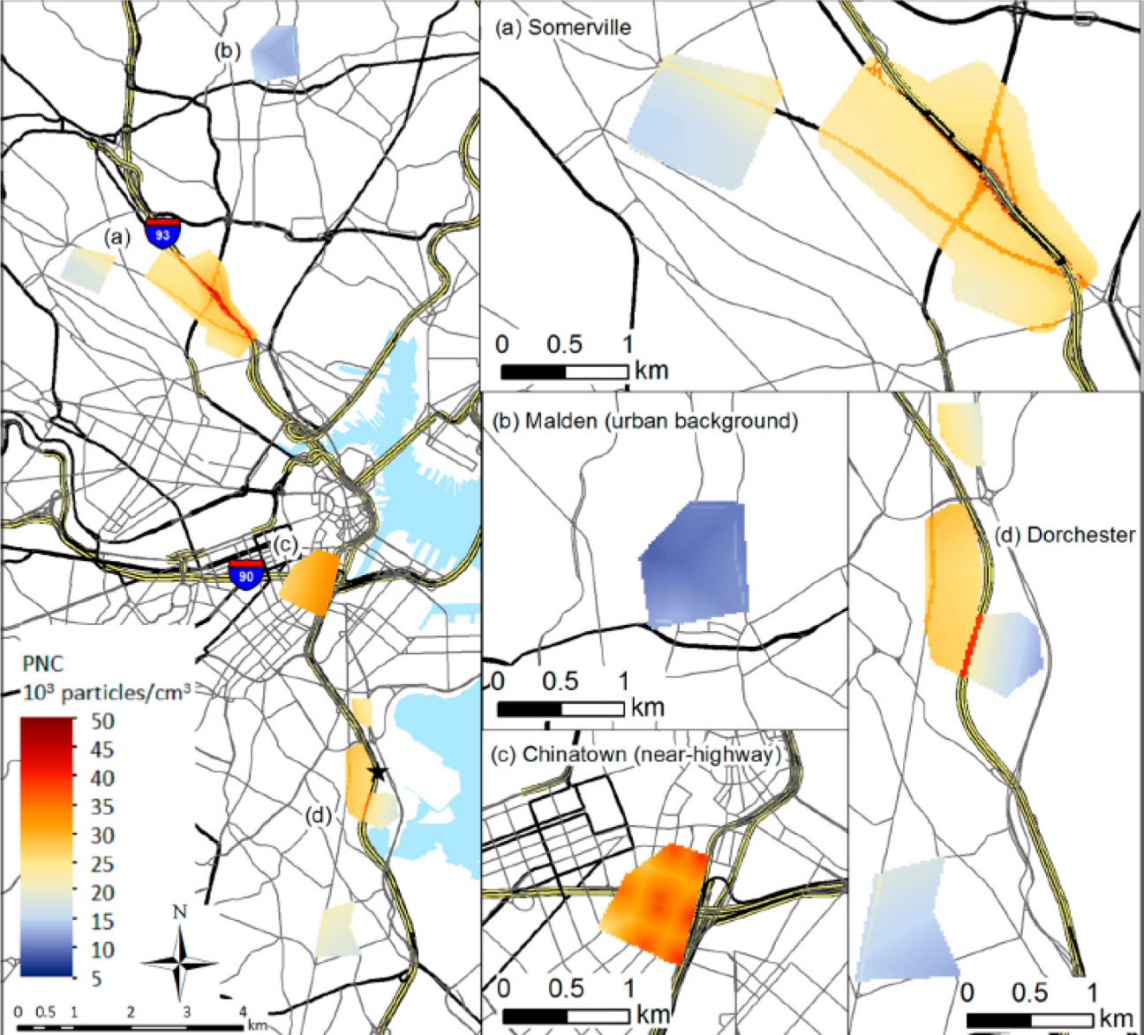
Ultrafine particle (UFP) level maps. The Community Assessment of Freeway Exposure and Health (CAFEH) study conducted mobile air pollution monitoring in three sets of paired neighborhoods, near the highway and >1 km from the highway (listed as (**a**–**d**) on the map). UFP were measured as particle number count using a condensation particle counter. The data from the mobile monitoring were used to develop land use regression (LUR) models of particle number count (or PNC, a measure of ultrafine particles) for each of the study areas. The LUR models had a resolution of 20 m and 1 h. The hourly estimates of PNC level were then averaged over a year to calculate the annual average values that are plotted in this figure.

This initial study was among the first to document the relationship between UFP exposure and health and was groundbreaking for its highly detailed attention to assessing exposure [15]. Figure 2: Near Roadway Exposures in Chinatown and Somerville, reprinted with the authors’ permission, is a map of the two communities illustrating the location of the highway and annual averages of UFP levels in each community; this map was previously printed in *Environmental Science and Technology* [16].

Over the years the partnership has grown, contributing substantially to the extant literature as it relates to near-roadway pollution, especially ultrafine particles [16–19]. Because of the CBPR framework in which community partners participate in the research decision-making, planning, implementation and dissemination, early findings have been used to inform the development of intervention studies designed to mitigate the health effects of near-roadway exposure [20]. Thus, the partnership has sought to translate both its own research and intervention studies as well as the broader relevant literature into policy and practice, seeking to guide community, municipal, regional and state responses to the problem.

Specifically, over the last 4–5 years the partnership has consciously sought to influence both community-level practices and policies, supported substantially in that effort by the Kresge Foundation ($775,000 over 4 years, total). The core of this funded effort was to engage the City of Somerville in developing an ordinance that would be protective for people living near the highways and major roadways in the city. However, and quite organically, these efforts ended up including many additional consultations on building design, the placement of parks, and other regulatory policy proposals. While the core focus was on Somerville, many of the ancillary consultations involved other municipalities.

## 2. Methods

We conducted a small-scale unfunded study focused on consultations conducted by the partnership. This study of the CAFEH partnership consultations was considered exempt by the Tufts University Social Sciences Institutional Review Board (Protocol #1,706,015) because the interviews collected professional knowledge instead of personal information. A total of 13 consultations were conducted by the CAFEH partnership, see Table 1.

Qualitative methods were used to explore how knowledge generated by the CAFEH partnership was translated through technical assistance consultations to inform local planning, practice and policy. Key informant interviews were conducted with individuals from entities (municipal, state and private). We specifically interviewed individuals for whom members of the CAFEH research partnership provided consultations to between 2013 and 2017.

### 2.1. Sampling and Recruitment

The sample included all individuals who participated in technical assistance consultations with members of the CAFEH partnership. The student researcher met with members of the project steering committee, which includes community and university partners. A list of individuals who participated in the consultations was generated (*n* = 13). Participants were contacted by email and the goal of the interview was explained. Individuals who agreed to participate in the interviews (*n* = 13) had interviews scheduled. At the time of the interview the researcher reviewed the goals of the study and procedures and assured interviewees that personal and organizational identifiers would not be reported.

### 2.2. Data Collection

The interviews explored four primary areas. First, participants were asked to describe their connection to the CAFEH partnership. This included how they learned about the partnership and came to seek technical assistance and consultations. Second, participants described their interactions with CAFEH, more specifically the nature of the consultation. In addition, questions explored the ways in which their consultation with CAFEH influenced their work and decision-making. Third, we explored the extent to which participants viewed integrating protective measures against traffic pollution as feasible and acceptable. Finally, we asked about the ways in which individuals were using and disseminating information they gained through their interaction with the CAFEH partnership (interview guide available upon request).

### 2.3. Data Management and Analysis

During the interview, the interviewer recorded responses by hand in as much detail as possible. After each interview notes were typed in a word document. The data was coded by question using thematic analysis [21]. A sample of the interviews were then reviewed by two researchers who thematically coded the content by question. This process involved the researchers reading the interview notes multiple times to immerse themselves in the data, exploring responses to each individual questions [21] and reflecting on their questions and reactions to the responses [22]. The researchers met and discussed the initial analysis to ensure consistency in their analytic approach [23]. They then developed codes by labeling and naming selected text segments. The initial codes were organized into themes and used to develop a code book, which was applied across the thirteen interviews. Of note, the codes were applied by one researcher and reviewed by the second. When coding was complete the researchers met to compare and reconcile codes. Quotes illustrating key themes were selected to produce a succinct and logical story identified from the data [21]. Themes and quotes were shared with a study team member (DB), who had been involved in all the consultations to further contextualize the findings.

## 3. Results

All the 13 individuals invited participated in interviews. Consultations were conducted by CAFEH partners with individuals from diverse sectors: public health leaders (2), developers (4), local (2) and regional (1) planners, state officials (2), a school leader (1) and a community leader (1).

### 3.1. Benefits of Consultation

All interviewees described their interactions with the CAFEH partnership as positive. Having access to research findings and evidence from the partnership was seen as a benefit. In many instances co-learning was described, in which interviewees were learning from the partnership and the research partnership was learning from them. This illustrates the importance of ongoing efforts to translate research in a way that allows the end users to further contextualize the findings.

“It’s been amazing to have learned from cutting-edge scientists and researchers. It also provided [us with] the benefit of making sure the research world understands what it’s like to be in resource management. It’s easy to fall in love with abstract solutions that aren’t feasible. We’ve been able to learn from each other—what does mass zoning law mean, what are the economics of real estate, what are traffic calming and engineering solutions. Always appreciated a pragmatic approach. It’s the application of the best science inspiring cutting-edge policy.” (I009)

Some developers reported receiving positive recognition for the changes they were able to make which were described as cost effective:

“The firm has to think about every aspect during the planning of developments to address every potential issue. … taking into account traffic-related air pollution and health is ‘what sets us apart’. We can explain to clients with more knowledge about the decisions [we] make regarding planning and what will be beneficial to the client and those using the space over the lifetime of the building.” (I002)

“The changes that needed to be made [to mitigate near-roadway pollution] were easy and led to positive press and positive recognition for the firm.” (I005)

Others, however, recognized the benefits of new knowledge, but were skeptical of the partnership’s ability to enact policy change. This was largely due to resource constraints and broader barriers that were both economic and political:

“It was informative. Does it create policy? Not really. [The data] does provide the information to think more about particulates and air pollution, but at the micro-policy level, with few resources, it doesn’t seem it will create change.” (I003)

### 3.2. Barriers to Change

Many respondents described barriers to mitigating near-roadway exposure through policy and practice. These included: cost, public will, politics and the availability of building space. In addition, participants described not knowing where to begin given the complexity of community level factors influencing local decision-making. Illustrative quotes from participants describing these barriers are provided in Table 2: Barriers to mitigating near-roadway exposure. Despite the barriers, most planners and developers agreed that, “generally speaking, if mitigation methods are factored in during early stages of development, it’s easier to resolve.”

### 3.3. Impacts of Consultation

The CAFEH partnership was able to achieve some meaningful translation impacts at multiple levels. All interviewees described the benefits of having access to empirical data and learning more about both the impacts of near-highways exposure as well as strategies for mitigating it. This is illustrated in the following quotes:

“Working with [researcher name and university name] is the best example of engaging with a research scientist and hearing research firsthand. It leads to policy we can enact.” (I006)

“This experience [hearing from the partnership] has increased knowledge and opened up the dialogue related to transportation-related air pollution.” (I004)

“During involvement with [university name], [I] learned quite a bit about traffic-related air pollution issues, the role of UFPs in the environment, their effect on health, and the implications of TRAP [traffic-related air pollution] within the school environment. This knowledge pushed [me] to improve ventilation, including the placement of fresh air intake and bumped up filtration used to MERV [Minimum Efficiency Reporting Value] 13 to provide best air quality in building.” (I002)

Participants described initially learning about the work of CAFEH through a variety of channels, such as newspaper articles, community meetings and word-of-mouth:

“[I] remember seeing a report by Chinatown Progressive Association in [the] Boston Herald opposing design of [the] school, which sited CAFEH research. The report was what first caught [my] attention and caused [me] to reach out to speak with [the investigator].” (1001)

In addition, participants were directly referred to the research team by collaborators, and in a few cases were contacted by the partnership as they began to develop intervention studies.

**Figure 3.**
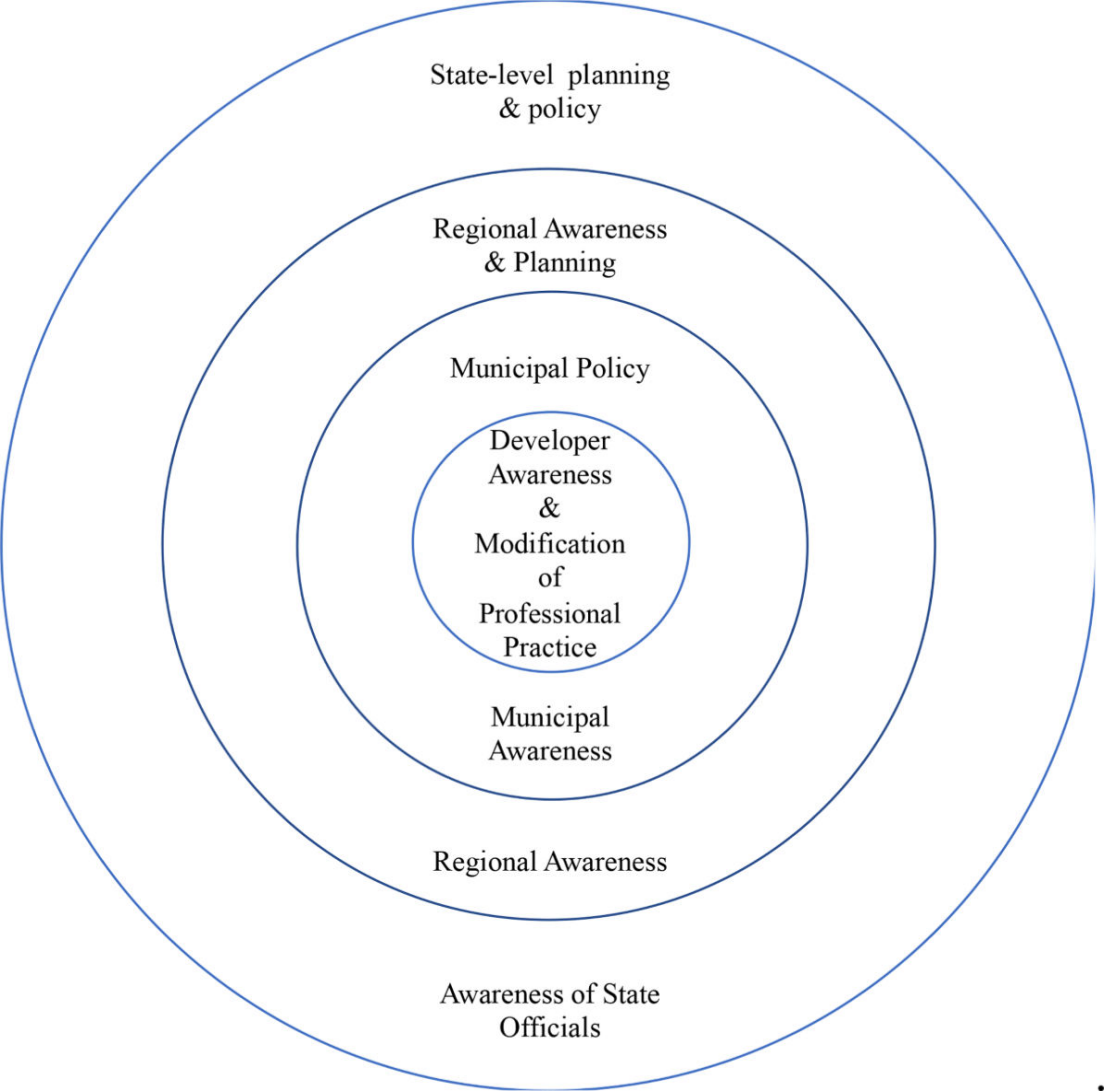
Levels of CAFEH impact.

Impacts have been seen at multiple levels, including developer behavior as well as municipal, regional and state policy-practice levels (see Figure 3: CAFEH Impact).

The most common impacts described were related to developer decisions about the type of filtration they choose. Developers from both Boston Chinatown and the City of Somerville have begun using higher levels of Minimum Efficiency Reporting Value (MERV) filtration.

“Prior to involvement with [university name], [we] were carrying standard filters and instead, it was proposed to use a more robust filter. Felt it was relatively easy thing to do. Asked general contractor to give a cost estimate for MERV 13 filters, which are typically only seen in hospitals and institutions. The $500 change order for a 30-million-dollar rehab was nothing. For a small amount of money, [we] were able to make a big impact in air quality.” (I005)

“[I] became informed of options I could explore regarding air purification and came up with a different filtration system from what was the original option. [We] chose a manufacturer with higher MERV rating.” (I008)

“[I] met [researcher name] four years ago at Charrette for a school being proposed in Chinatown near highways where they were talking about air quality and impact … [I] started thinking of ways to improve the air quality in the community health center in a high rise situated near the highway ramp. Discussed introducing a higher rated MERV filter.” (I010)

Through engagement with municipal planners, public health leaders and school leaders CAFEH has also increased knowledge and informed municipal practice and policy. Table 3: Municipal Impacts highlights illustrative quotes from three cases describing the impact of the CAFEH partnership at the municipal level.

Meanwhile, at the regional and state levels the CAFEH partnership has led to the prioritization of near-roadway pollution as a public health priority. For example, a quasi-governmental planning agency which, advocates for municipal policies across the region to consider potential health impacts, added near-roadway pollution as a priority after engaging with the CAFEH partnership.

“Prior to involvement with [the partnership], our work was focused on how to build healthy neighborhoods, design transportation systems, and construct parks, with the primary focus on physical activity and healthy eating, and not as involved on the environmental health side. This experience has increased knowledge and opened up the dialogue related to transportation-related air pollution…. In 2013–2014, taking air pollution into consideration became standard practice and was built into the health impact assessment. Air-pollution-related health assessments are now built into all housing and transportation needs/plans. Now, [we are] starting to incorporate the same type of assessment into plans for parks and open spaces.” (I004)

At the state level, knowledge generated by the CAFEH partnership inspired the introduction of legislation designed to require protective measures in housing and schools built close to highways. In addition, it resulted in the team being able to further disseminate information through legislative briefings.

“[I] have filed bills based on studies coming out of CAFEH and sponsored legislative briefings. [In addition, I] have asked [CAHEH team members] to do presentations for legislators on air quality and health. [I also,] had a Transportation Planning Director from the Netherlands come and give a talk about getting traffic out of urban centers.” (I007)

Overall, participants reported that what they learned had continued to influence their decision-making long after their interaction with CAFEH.

## 4. Discussion

We explored the extent to which research associated with the CAFEH partnership was translated to informing planning, policy and practice by individuals who received technical assistance consultations from our project. The literature indicates engaging community stakeholders and residents in the research process, from planning and implementation to dissemination, facilitates research translation [3,24]. Doing so creates a common way of talking about and understanding research [25]. CBPR is designed specifically to move research to practice and policy [26]. For example, in Houston a CBPR partnership including residents as well as stakeholders from the academy, government and industry and focused on metal emissions was able to increase capacity for change by creating a shared understanding of the issue across stakeholder groups [27]. This process led to the development of a comprehensive community action plan [27].

Perhaps the greatest success of our partnership was not so much policy and planning but the diffusion (or communication) of knowledge [28], which is an important first step in planning and policy change. CBPR efforts in New York led to important environmental policy changes over time but initial steps involved increasing awareness [29]. Of note, the West Harlem Environmental ACTion, Inc. (WE ACT) and Columbia University were able, like us, to generate a clearly defined problem statement and create broad awareness across sectors of the issue which were important first steps in moving to agenda setting [29]. In our case, partnering with community members increased interest in our project among other key stakeholders in the community and enhanced our ability to communicate finding to these stakeholders. This happened as stakeholders learned about our work through local dissemination efforts led by research and community partners as well as through community partner networks. Interviewees learned about the research locally and that is what led to technical assistance consultations.

These initial consultations allowed us to begin to: (1) ensure the problem was clearly and consistently defined and (2) increase awareness among diverse stakeholders. Knowledge generated by our project and disseminated locally through technical assistance consultations informed thinking and practice. Community partners facilitated researcher access to local decision makers, which influenced decision making related to practice. This was most clear in the case of MERV filtration in buildings and homes. We were also able, through consultations, to develop relationships needed for policy advocacy in the future.

In California, community partnerships have been able to: (1) inform local legislation, (2) elevate health in the context of transportation policy, and (3) delay transportation projects until health impacts can be explored, and (4) the partnership also led to an increase in community member voices in the decision-making process [30].

Although we are not yet there in Massachusetts, our partnership has begun to lay the foundation by increasing awareness. More specifically, through technical assistance consultation we were able to translate scientific knowledge into understandable concepts that could be digested by local stakeholders to inform their practice [31]. In the literature, the prevention synthesis and translation system is a key component of the interactive systems framework which is designed to disseminate and implement innovation [31]. Local decision-makers have become more aware of the impacts of near-roadway exposure on health.

This study is not without limitations. Ideally, a case study design would have facilitated our ability to learn more about the nature of consultations as well as their impact. However, data was not available, as consultations were not part of the initial protocol but rather an outgrowth of local dissemination that emerged organically over the course of the project. As such, we relied on key informant interviews. Similarly, interviews were conducted with individuals; as such, the extent to which knowledge from the consultation impacted the organization is unclear, as is the extent to which the individual championed the information gained. It may be that other individuals at the organizations and institutions interviewed have alternative views, although the interviews were chosen for people who were the primary recipients of CAFEH consultations.

Future research should explore knowledge of the health implications of near-roadway pollution in organizations beyond those who have directly interfaced with the CAFEH teams. In addition, policy change takes time, and it is still fairly early in the life of the partnership, so the full impact may not be realized for years. This highlights the importance of long-term follow-up. Nonetheless, we have seen benefits with respect to translation, and since engaging in this exploratory study we have applied for and been awarded grants associated with technical assistance consultations, including a Near Highway Research to Action grant from the National Institute for Environmental Health Sciences.

## 5. Conclusions

The CAFEH partnership has demonstrated that community dissemination can contribute to deeper community engagement and facilitate translation. Technical assistance consultation that resulted from community dissemination efforts facilitated translation by informing local decision-making and practice. Community research partnerships can link diverse stakeholder groups to empirical evidence, bridging research to both policy and practice. For CAFEH, local dissemination efforts led by community partners was critical in this effort in that it led to further community engagement and consultation. If we are going to truly shift to a health in all policies model there is a need for strategies to bridge the private, public and health sectors. Community research partnerships are an important vehicle for this work.

## Figures and Tables

**Table 1.** Consultations.

#	Activity Description
1	Consulting with an architect on building design and ventilation for a new construction near highway school.
2	Consulting with a headmaster on building design and ventilation for a new construction near highway school.
3	Technical assistance, including air monitoring for design of a largely outdoor community art space near a highway.
4	Advising a regional agency about their recommendations for municipalities with respect to traffic pollution.
5	Advising about modifications to ventilation and filtration in an existing housing development.
6	Consulting on building design and ventilation for new construction of housing near a highway.
7	Technical assistance for developing state legislation.
8	Consulting on building design and ventilation for new construction of housing near a highway.
9	Technical assistance, including air monitoring, to a municipal agency about urban planning for a high traffic neighborhood anticipating rapid redevelopment.
10	Advice about ventilation and filtration for a near highway new construction community room.
11	Technical assistance for a municipal public health agency about reducing risks from traffic pollution.
12	Technical assistance, including air monitoring, to a municipal agency about urban planning for a high traffic neighborhood anticipating rapid redevelopment.
13	Public comment and engagement with a state agency about promoting developments near major highways.

**Table 2.** Barriers to mitigating near-roadway exposure.

	Themes and Illustrative Quotes
Place	*“Tight urban sites, where high density of traffic is surrounding the site, are substantially more difficult in terms of managing.” (I002)* *“The developer was not willing to stop development altogether or reorient [the] site. The site is on a hill that goes down to the highway. They were asked to cluster buildings and outdoor space more towards* *[street name] rather than [the] highway but weren’t able to for various reasons.” (I006)*
Politics	*“So far, none of [the] bills have been enacted due to tremendous pushback from realtors and [the] development community, because it makes it harder to build affordable housing, and also from libertarians who believe the government shouldn’t have the authority to tell people where to live. It’s a long horizon to achieve good outcomes.” (I007)*
Complexity	*“* … *siting of a residential building away from highway isn’t always possible. It’s necessary to employ individual protective measures. It’s a complex system, and there are lots of areas where things can break down.” (I011)* *“People are completely receptive to the research, but the trouble comes from how to make it work.” (I012)*
Cost	*“There are a lot of things one could do, but [they] would not necessarily be financially viable.! (I008)* *“Money is an obstacle. If money were more available, they could incorporate other protective measures, such as a filtration system.” (I003)*

**Table 3.** Municipal impacts.

	Illustrative Quotes by Municipal Sector
Schools	*“[I] wanted to make sure the building would be safe for students, faculty and myself, so [I] got involved with [the] design team, citing [university name] research. Involvement with [university name] definitely impacted the design of the school. One method used to lower UFP exposure was to increase MERV filter rating.” (I001)*
Public Health Board/Commission	*“[I] consulted with [researcher] regarding his early research on traffic-related air pollution.* *During this time, the [Board of Health] BOH was in negotiations with [developer name] Residential, LLC regarding the [town name] 40B project, a proposed 300-unit residential development near a stretch of [Route] 128. Being the first large-scale development, and the first so close to the highway, the town opposed the development and tried to put up barriers. At this time, the BOH was asked to weigh in, and [CAFEH research] was [presented]. The developer sued the town, and the developer and BOH were told to resolve differences. BOH wouldn’t settle because of potential health effects on future residents. In 2016, [CAFEH researcher] came in again and talked to the BOH about possible mitigation measures and what steps the board could take through enacting regulatory powers or negotiating with developer. The BOH negotiated with the lawyer and VP of development to make the necessary changes to mitigate the health risks of the future residents.” (I006)*
Municipal Planners	*“Those on the housing team started sharing more info with staff on health impacts for people living near highways. [We] wanted to ensure concerns coming out of study were being considered [by developers]. [We] have control over when someone builds something new* … *[so], with a couple of projects near highways [we required] getting discretionary review from planning board.” (1012)*
